# MALDI-TOF MS Biomarkers for Methicillin-Resistant *Staphylococcus aureus* Detection: A Systematic Review

**DOI:** 10.3390/metabo15080540

**Published:** 2025-08-08

**Authors:** Pedro Santos, Irina Alho, Edna Ribeiro

**Affiliations:** 1 Escola Superior de Tecnologia da Saúde, Instituto Politécnico de Lisboa (ESTeSL–IPL), Av. D. João II, Lote 4.69.01, Parque das Nações, 1990-096 Lisboa, Portugal; pmisantos@ulsac.min-saude.pt; 2 Serviço de Patologia Clínica–Unidade Local de Saúde do Alentejo Central, Largo Senhor da Pobreza, 7000-811 Évora, Portugal; 3 Faculdade de Medicina, Universidade de Lisboa, Av. Professor Egas Moniz, 1649-028 Lisboa, Portugal; ialho@medicina.ulisboa.pt; 4 Health & Technology Research Center (H&TRC), Escola Superior de Tecnologia da Saúde, Instituto Politécnico de Lisboa, Av. D. João II, Lote 4.69.01, Parque das Nações, 1990-096 Lisboa, Portugal

**Keywords:** MALDI-TOF MS, *Staphylococcus aureus*, methicillin-resistant *Staphylococcus aureus*, biomarkers, discrimination, artificial intelligence

## Abstract

Background/Objectives: Methicillin-resistant *Staphylococcus aureus* (MRSA) infections remain a significant challenge in healthcare. Conventional and molecular techniques used for MRSA identification are either time-consuming or costly. Alternatively, Matrix-Assisted Laser Desorption/Ionization Time-Of-Flight Mass Spectrometry (MALDI-TOF MS) offers a rapid method for microbial identification and has the potential to detect biomarkers that distinguish methicillin resistance in *S. aureus* isolates. The aim of this study was to identify methicillin-resistant discriminative biomarkers for *S. aureus* obtained using MALDI-TOF MS. Methods: A systematic review was conducted by searching databases such as PubMed and Web of Science for studies that focused on MRSA detection with biomarkers by MALDI-TOF MS, including all relevant studies published up to July 2024. The review protocol was registered in PROSPERO registry. Results: A total of 15 studies were selected for analysis. Data were extracted on study location, sample size, MALDI-TOF MS analyzer, sample preparation, methicillin resistance and sensitivity biomarkers, and the use of Artificial Intelligence (AI) models. Notably, PSM-mec and delta toxin were frequently reported as informative biomarkers, detectable at 2414 ± 2 Da and 3006 ± 2 Da, respectively. Additionally, eight studies used AI models to identify specific biomarkers differentiating methicillin-resistant and methicillin-sensitive strains, based on differences in peak intensities or the exclusive presence of certain peaks. Moreover, two studies employed detection of MRSA in low concentrations from biological samples and others employed an optimized matrix solution for improved analysis. Conclusions: Overall, MALDI-TOF MS is not only a powerful tool for the identification of bacterial isolates but also shows strong potential for rapid, cost-effective detection of methicillin resistance in *S. aureus* through biomarker analysis. Given that it is already implemented in several clinical laboratories, this approach could be adopted without significant additional cost.

## 1. Introduction

Methicillin-resistant *Staphylococcus aureus* (MRSA) infections remains one of the top priority bacterial agents, according to the World Health Organization, due to its continued role as a challenging infectious agent, responsible for infections that inflict a high financial burden on health services [1,2]. MRSA infections are associated with higher mortality and morbidity compared to methicillin-susceptible *S. aureus* (MSSA) infections [2,3,4]. Therefore, rapid and accurate detection and differentiation of MRSA from MSSA becomes a priority to determine appropriate antimicrobial treatment [5].

Currently, conventional techniques for microbial identification and microbial resistance detection rely on phenotypic methods, such as the Kirby–Bauer test, broth dilutions, and E-test [6,7,8]. Although automated systems are broadly used in microbial laboratories, the turnaround time typically ranges from 48 to 72 h to identify the most suitable antimicrobial [6,7,9,10]. To shorten this timeframe, molecular techniques, such as Real Time-Polymerase Chain Reaction (RT-PCR) have been used, reducing detection time to approximately 2 h [2,6,11]. However, these methods, both classical microbiological protocols and, particularly, molecular biology techniques have some limitations, including the need for different materials and reagents, different trained professionals, several specific equipment’s are highly expensive and it is time-consuming, which increases the financial burden and delays in patient’s diagnosis [2,6,9,11].

The WHO defines diagnostic stewardship as “coordinated guidance and interventions to improve appropriate use of microbiological diagnostics to guide therapeutic decisions. It should promote appropriate, timely diagnostic testing, including specimen collection, and pathogen identification and accurate, timely reporting of results to guide patient treatment” [12]. The development of faster methods for microbial diagnosis aims to shorten the susceptibility timeframe to infection, limit the use of presumptive antimicrobial therapy, reducing the risk of the development of antimicrobial resistances, and expedite the implementation of appropriate antimicrobial therapy [9,12,13].

MALDI-TOF MS is a technology that is used to identify microbiological isolates by analyzing the Peptide Mass Fingerprint (PMF) [13,14,15,16]. The PMF is generated from macromolecules, primarily ribosomal and housekeeping proteins, and compared to the Main Spectra Profile (MSP) stored in extensive and up-to-date databases [13,14,15,16,17]. Turnaround time for identification of microbial isolates with this method can take around 5 to 45 min, depending on whether protein extraction is required—though this step has become less necessary due to improvements in method efficiency [13,14,15,16,18].

Beyond identification, MALDI-TOF MS also has the potential to detect antimicrobial resistance through specific biomarkers [13,15,16,19]. This can be particularly useful in distinguishing methicillin resistance, based on differences in the PMF between MRSA and MSSA isolates, without requiring additional sample preparation or incurring extra costs [20,21,22,23]. Phenol-soluble modulin peptide (PSM-mec) has a mass/charge (*m*/*z*) around 2414 Daltons (Da) and delta toxin with 3006 Da have some diagnostic value yet limited [24,25,26,27,28]. Additionally, through the observation of the spectral mass, there are other discriminative biomarkers, that have differences in intensity or do not coexist in the PMF of MRSA and MSSA isolates, allowing detection of a greater portion of MRSA isolates [21,22,29,30]. Furthermore, integrating MALDI-TOF MS with advanced mathematical and computational models, such as AI-based approaches, have been shown in several studies as an important tool for rapid discrimination of MRSA and MSSA in laboratory diagnosis [31,32,33,34]. Also, optimization of matrix solutions and the development of protocols for detecting MRSA at low concentrations directly from biological samples may further improve diagnostic performance [35,36].

Here we aimed to perform a systematic review to determine which biomarkers could be used to detect and discriminate between MRSA and MSSA isolates using MALDI-TOF MS technology.

## 2. Materials and Methods

### 2.1. Study Design

This systematic review was conducted following the guidelines defined by the Preferred Reporting Items for Systematic Reviews and Meta Analyses (PRISMA) from 2020 [37]. The protocol was registered in the beginning of the screening process in the PROSPERO registry, under the record number CRD42024591404.

### 2.2. Eligibility Criteria

The inclusion criteria involved studies addressing MRSA and MSSA human infections and the identification of biomarkers of *S. aureus*. Studies involving veterinary infections, infections by other infectious agents, and sole identification of isolates by MALDI-TOF MS without analyzing biomarkers were excluded. Also, all available studies with full-text access and redacted in English language that appeared in the collection process on databases were considered eligible. The systematic literature reviews and meta-analyses were only used as check references. Articles not fulfilling the eligibility criteria were not subjected to additional review (Table 1).

### 2.3. Search Strategy and Database Search

A comprehensive search of the PubMed and Web of Science databases was conducted to identify relevant original studies published up to 27 July, 2024. The following search terms were used: (MALDI-TOF) AND ((MRSA) OR (methicillin-resistant *Staphylococcus aureus*) OR (methicillin resistant *Staphylococcus aureus*) OR (methicillin resistant *S. aureus*) OR (oxacillin resistant)) AND ((MSSA) OR (methicillin-sensitive *Staphylococcus aureus*) OR (methicillin sensitive *Staphylococcus aureus*) OR (methicillin sensitive *S. aureus*) OR (oxacillin sensitive)).

### 2.4. Study Selection and Data Extraction

All retrieved records were organized using a reference manager (EndNote 21™, Clarivate Analytics, Philadelphia, PE, USA) and exported to a spreadsheet (Excel^®^ 2021, Microsoft Office, Washington, DC, USA). All studies resulting from the literature search were selected by title, abstract, and full-text based on the inclusion and exclusion criteria. Two independent reviewers conducted the selection process. Any discrepancies or disagreements were resolved by a third reviewer who reviewed the abstracts and full-texts to reach a final decision.

To maintain a coherent data extraction, relevant data collected from the final selected studies were organized in an Excel^®^ spreadsheet by Microsoft Office, which included the following: author and year, sample size, study location, reference method, MALDI-TOF analyzer, sample preparation, MRSA and MSSA biomarkers, and the use of AI models.

### 2.5. Risk of Bias Assessment

The quality of the studies, the risk of bias and the accuracy of the data were assessed using the Joanna Briggs Institute’s critical appraisal tools, according to the study design (case–control studies, cross-sectional studies, etc.) [38]. The studies were evaluated against their methodological quality, possibility of bias in its design, conduct, and analysis. The risk-of-bias VISualisation (robvis) tool (https://mcguinlu.shinyapps.io/robvis/ [accessed on 10 June 2025]) was used to generate a graphical representation and summary of the risk of bias [39].

All 15 studies included in this systematic review had a cross-sectional design and were evaluated using an 8 questions checklist from Joanna Briggs Institute, answered with “Yes, No, Unclear or Not Applicable” [40] (Table A1). The results obtained are presented in Table A2. In question 3, studies that did not use gold-standard method to detect methicillin resistance were classified as “No” and studies that did not clearly state *mecA* detection were classified as “Unclear” [22,25,27,34,35]. In question 6, all studies were classified as “Not Applicable”, due to the nature of the selected studies that did not require strategies to deal with effects of confounding factors [21,22,24,25,26,27,28,29,30,31,32,33,34,35,36]. In question 8, one study was classified as “Unclear”, because it did not clearly perform a statistical analysis [33].

In order to present a visual representation of the data using risk-of-bias VISualization (robvis) tool, it was necessary to convert the reported answers according to the level of risk of bias: ‘Yes’ into ‘Low’, ‘No’ into ‘High’, and ‘Not Applicable’ into ‘No Information’. The “Yes” answer were assigned with 1 point and “No”, “Unclear”, or “Not Applicable” answers were assigned with 0 points.

**Figure A1:**
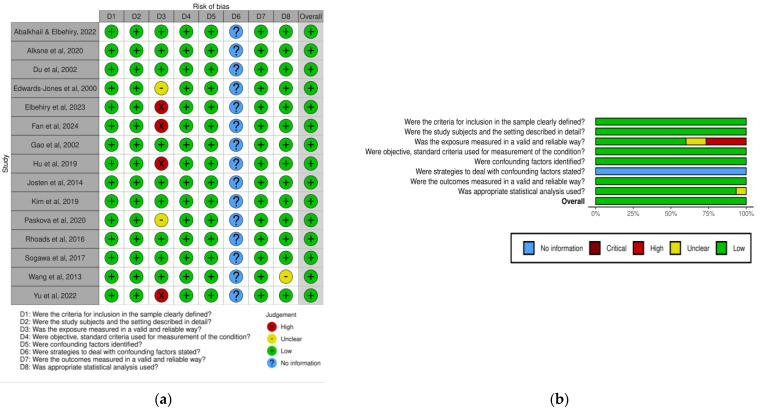
Risk of bias graph of cross-sectional studies: (**a**) traffic light plot, (**b**) summary plot. Red symbol corresponds to a high risk of bias, yellow symbol corresponds to unclear risk, green symbol corresponds to low risk, and interrogated blue symbol corresponds to lack of information [21,22,24,25,26,27,28,29,30,31,32,33,34,35,36,39].

Overall risk of bias was categorized as low when the study attained more than 70% of ‘yes’ ratings, moderate for 50–69%, and high for less than 49%. The individual articles scored between 75% and 87.5%. As a result, the overall quality of the studies and accuracy of the data was good, and the risk of bias was low. The graphical and summary illustration of the risk of bias is shown in Figure A1.

### 2.6. Statistical Analysis

Statistical analysis was performed using descriptive statistics and data processing were performed using Microsoft Office Excel^®^ software. For some studies that did not clearly display the differentiation accuracy, we determined it using the MedCalc^©^ Software version 23.0.8, Ostend, Belgium [41]. To determine the accuracy, we used the “Diagnostic test evaluation calculator” with the following data: the number of cases in the diseased group that tested positive and negative, and the number of cases in the non-diseased group that tested positive and negative [41]. Diagnostic accuracy depends on the disease prevalence. Since the prevalence of MRSA worldwide is variable and the studies did not perform prevalence calculations, we used the mean accuracy results in these studies.

## 3. Results

**Figure 1:**
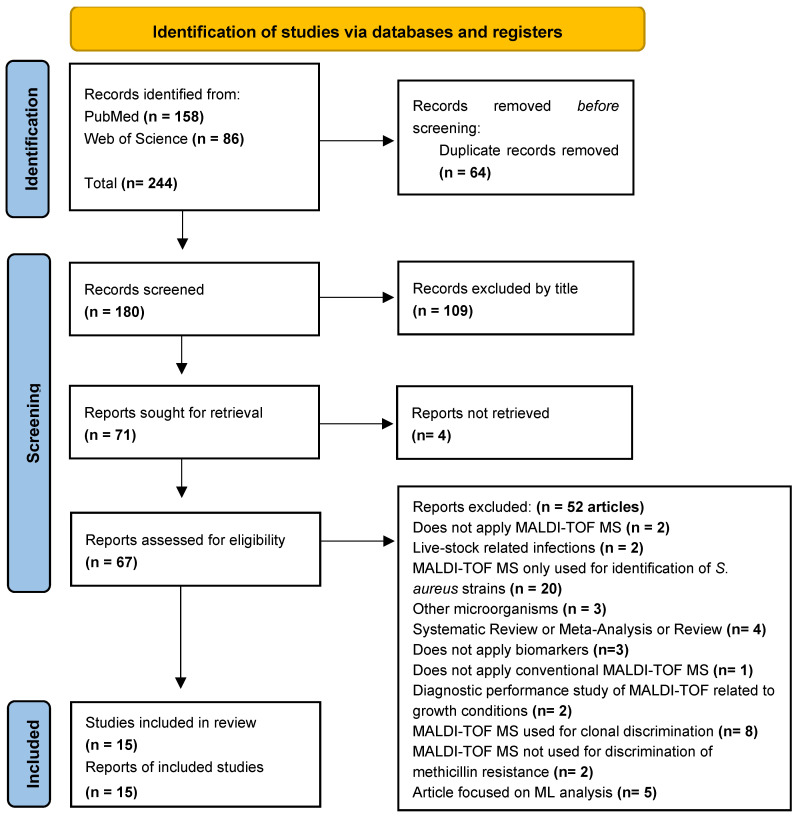
PRISMA-based selection of articles. Page, M.J., McKenzie, J.E., Bossuyt, P.M., Boutron, I., Hoffmann, T.C., Mulrow, and C.D. et al. (2021). The PRISMA 2020 statement: an updated guideline for reporting systematic reviews. *BMJ*, *372*, n71. https://doi.org/10.1136/bmj.n71 [37]. From http://www.prisma-statement.org/.

For this systematic review, 244 articles were found, of which 158 were retrieved from PubMed and 86 from Web of Science databases. After duplicate removal using EndNote™, 64 articles were excluded, with 180 unique studies remaining. After screening by title, we excluded 109 studies, with 71 remaining for abstract and full-text screening. Through careful consideration regarding eligibly criteria, we excluded a total of 56 studies, from which 4 studies could not be retrieved due to a lack of an abstract or full-text. The others were excluded due to the following reasons: did not apply MALDI-TOF MS (n = 2); livestock related infections (n = 2); MALDI-TOF MS only used for identification of *S. aureus* strains (n = 20), other microorganisms (n = 3); systematic review or meta-analysis or review (n = 4); did not apply biomarkers (n = 3); did not apply conventional MALDI-TOF MS (n = 1); diagnostic performance study of MALDI-TOF MS related to growth conditions (n = 2); MALDI-TOF MS used for clonal discrimination (n= 8); MALDI-TOF MS not used for discrimination of methicillin resistance (n = 2); articles focused on Machine Learning (ML) analysis (n = 5). At the end, we selected 15 studies in this systematic review (Figure 1).

### 3.1. Selected Studies

The collected data from the included studies is summarized in Table 2, which contains the following information: author and year, sample size, study location, reference method, MALDI-TOF analyzer, sample preparation, MRSA and MSSA biomarkers, and the use of AI models. Most studies used MALDI-TOF MS diagnostic systems from Bruker Daltonics and bioMérieux. Samples commonly underwent preparation using the on-target protein extraction method (O-PEM) and reference and/or gold-standard methods, such as the Kirby–Bauer test and PCR detection of the *mecA* gene, were employed to confirm methicillin resistance. More than half of the studies used an AI model, mainly Machine Learning (ML) algorithms, to identify or assess potential biomarkers (n = 8). Sample sizes varied considerably across studies, ranging from 14 to 20,359 *S. aureus* isolates. Regarding the geographic distribution, more than half of studies originated in the Asian continent (n = 9).

### 3.2. PSM-Mec and Delta Toxin

PSM-mec is encoded by the *psm-mec* gene, which is present in isolates containing Staphylococcal Cassette Chromosome mec (SCCmec) types II, III, and VIII [24,25,26,27,28]. It consistently exhibits a mass-to-charge (*m*/*z*) around 2414 Da, with minimal variability within the uncertainty interval [24,25,26,27,28]. PSM-mec has been described as a highly specific biomarker, with 100% specificity, indicating methicillin resistance not only in *S. aureus* but also in other Staphylococci, namely *S. epidermidis* [24,25,26,27,28]. However, its sensitivity was described as low and with variable reproducibility/repeatability, potentially identifying up to half of MRSA isolates (Table 3) [24,25,27]. Also, its detection could only be attained through intact cell method (ICM) and O-PEM protocols [24,26,28].

Delta toxin is encoded by the *agr* gene, which also regulates *psm-mec* gene expression and has a reported *m*/*z* of around 3006 Da, with little variation [26,27,28]. An exception was noted in clonal complex 1 (CC) isolates, described by Josten et al. (2014) which showed a *m*/*z* of 3037 Da [26]. Detection using ICM and O-PEM protocols was found to be more effective than the in-tube protein extraction method (I-PEM), particularly for isolates with SCCmec II and III [24,26]. Thus, yielding higher signal intensity, compared to the PSM-mec biomarker [24,26]. Nevertheless, delta toxin is not specific to MRSA, as it may be present in any *Staphylococcus* spp., and thus considered as an auxiliary biomarker to PSM-mec [24,26].

### 3.3. Additional MRSA and MSSA Biomarkers

Additional biomarkers associated with methicillin resistance and methicillin sensibility in *S. aureus* strains were identified in the remaining studies, through the analysis of divergences in the PMF between MRSA and MSSA isolates. These differences in the MALDI-TOF MS results could be assessed visually or analyzed using a data processing software. In most cases, these divergences were detected with the support of AI models, which facilitated the identification of biomarkers with differences in intensity or that do not coexist in the PMF of MRSA and MSSA isolates [21,22,29,30,31,32,33,34]. Reportedly, the studies included the following AI models: Principal Component Analysis (PCA), genetic algorithm, decision tree model, non-linear Support Vector Machine (NL-SVM), and Light Gradient Boosting Machine (LightGBM) [21,22,29,30,31,32,33,34]. Since these models relied on a larger number of biomarkers for identification of resistant and sensitive strains, they demonstrated improved performance in distinguishing between strains.

In studies that did not display the differentiation accuracy we used MedCalc^©^ Software version 23.0.8. Here we extracted from each study the number of cases in the diseased group that tested positive and negative, and the number of cases in the non-diseased group that tested positive and negative. As an exception, Wang et al. (2013) neither provided the accuracy nor clear sufficient data to allow a robust calculation of accuracy to be performed using this tool [33]. Therefore, we were unable to calculate the accuracy in this study. Additionally, the studies did not provide local data on the prevalence of MRSA, and due to the variable distribution of prevalence worldwide, we were not able to account for this variable in the accuracy calculations.

All of the studies included in Table 4 used an AI model to identify additional biomarkers, with the exception of Du et al. (2002) and Edwards-Jones et al. (2000) [29,30]. In these two studies, the authors collected a smaller *S. aureus* sample and the differentiation between MRSA and MSSA biomarkers was performed either through cluster analysis or through visual observation of mass spectral profiles [29,30].

The additional biomarkers identified through the analysis of mass spectra using AI models had an identification rate of between 78% and 100%. In the study by Sogawa et al. (2017), the accuracy ranged from 89% to 100% when three distinct groups were used, each with a sample size of between one hundred and five *S. aureus* isolates [32]. In the sole case of Yu et al. (2022), the area under the curve (AUC) presented by the authors ranged between 0.78 and 0.91, since the evaluation of the performance was measured in two distinctive internal and four distinctive external sample sets, with a gross total of 20359 *S. aureus* isolates [34]. The overall accuracy or AUC for the differentiation of MRSA and MSSA was reported to be greater than 90% in these studies (Table 4).

**Figure 2:**
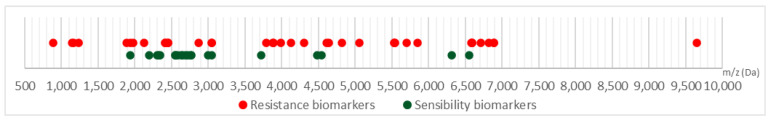
Distribution of MRSA and MSSA biomarkers through the mass spectra.

**Figure 3:**
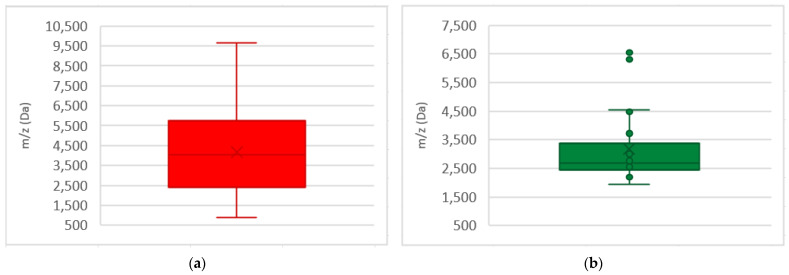
Box-plot representation of the distribution of biomarkers in *S. aureus*: (**a**) box-plot for distribution of MRSA biomarkers; (**b**) box-plot for distribution MSSA biomarkers.

**Figure A2:**
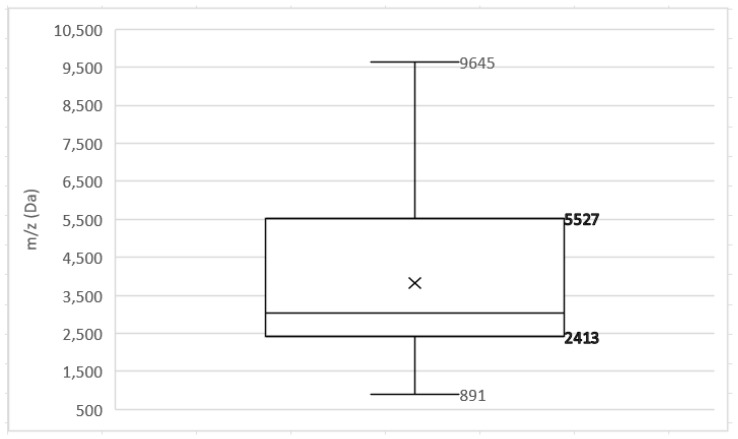
Box-plot for total distribution of the collected biomarkers.

We compiled all additional biomarkers to visualize their distribution throughout the mass spectra of both MRSA and MSSA biomarkers (Figure 2 and Figure 3). It can be observed that MRSA biomarkers were more numerous and displayed a more diffused distribution pattern (Figure 3a), while MSSA biomarkers were fewer and predominantly clustered between 2500 and 3500 Da with some outliers between 6300 and 6600 Da (Figure 3b). In this systematic review, the collected biomarkers were concentrated around 2400 and 5500 Da (Figure A2).

In addition to the studies focusing on additional biomarkers, one study employed magnetic separation for detecting MRSA in low concentrations in biological samples and another used an esterified CHCA matrix solution that enabled detection of hydrophobic peptides not detectable with conventional CHCA matrix preparation [35,36]. Both studies stated high efficacy in identifying or differentiating MRSA from MSSA strains (Table 4) [35,36].

## 4. Discussion

MRSA is a challenging pathogen found in nosocomial, livestock, and community environments, and it poses a high financial burden on health systems and with associated higher mortality and morbidity rates compared to MSSA infections [1,2,3,4,42]. Early detection and discrimination between MRSA from MSSA are critical to initiate targeted antimicrobial therapy and reduce the infection susceptibility window [2,43,44,45].

Currently, conventional microbiological identification and antimicrobial susceptibility testing typically rely on phenotypical and biochemical methods, which may take between 48 and 72 h [6,7]. Molecular techniques such as PCR methods allow for more rapid detection of methicillin resistance in *S. aureus*, reducing turnaround time to 2 h [6,7]. However, these methods may yield false-negatives, particularly when other *mec* genes are present or target amplification is insufficient [5,6,11]. A recently published hierarchical Bayesian meta-analyses was used to assess pathogen-specific resource which stated that there is an adjusted excess length of hospital stay estimated for MRSA with associated high financial costs [46]. Considering that the current applied methods, namely classical microbiological protocols and molecular biology techniques, are highly expensive and time-consuming, which increases the financial burden and delays patients’ diagnostics, the use of faster and accurate methods could particularly benefit lower income countries [2,6,9,11].

MALDI-TOF MS has become a significant advancement in healthcare as a tool for microbiological identification, providing reduced time of operation, laboratorial diagnostic report, and financial cost [13,14,15]. More recently, this technology has attracted attention in the antimicrobial resistance profiling, namely in the detection of MRSA biomarkers [9,14,16,19,20]. Since it is already widely employed in microbiology laboratories to identify microbiological isolates due to its well-known convenience and performance, investigating its potential for use in antimicrobial resistance detection without further costs or consumables, may enhance its practical value [9,14,16,19,20].

In this systematic review, we included studies using different instruments, experimental protocols and analytical approaches. Variations in equipment systems, database configuration, and application of AI models led us to focus on experimental outcomes, particularly in biomarkers detection in PMF. However, the protein extraction protocol significantly influences biomarker detection [18,24]. Chen et al. (2023) performed a meta-analysis which evaluated the performance between ICM and PEM and concluded that sensitivity is fairly the same, but ICM displays better specificity [23]. Our review further specified the level of PEM performed in the selected studies, into I-PEM and O-PEM, as they display different procedures and capability for microbial detection [18,47]. The confidence values between protocols are not very divergent, allowing for an accurate identification [18,47]. At least for microbial identification, it has become less common to perform protein extraction protocols, leading to simpler methods like ICM to achieve high accuracies, due to the improvement in the efficiency of the method [9,13,48]. Taking into account the possibility of the varied chemical nature of the biomarkers, we did not explore all the details of the influence of the protocol. For now, it may not be possible to develop standard protocols for sample preparation regarding biomarker detection. A more proficient way to achieve reproducible results would be to optimize biomarker detection with a given sample preparation protocol and introduce Data Science professionals or laboratory technicians who have been trained to use AI tools in laboratory diagnosis.

The biomarkers identified in our review were predominantly concentrated between 2400 and 5500 Da (Figure A2), consistent with findings in other studies [21,22,31]. Also, several authors found that within this interval there are strong intensity peaks that may be useful to differentiate between MRSA and MSSA strains [21,22,31]. The uneven distribution of MRSA and MSSA biomarkers (Figure 2 and Figure 3) might be due to greater number of resistance biomarkers available in the selected studies, rather than to higher protein expression of MRSA compared to MSSA stains [49,50]. Additionally, the predominance of studies from Asia may influence biomarker variability, reflecting the known geographics diversity of *S. aureus* [4,51]. In fact, several MRSA clones have emerged and disseminated globally, with enormous geographic prevalence variability that can range from 0.6% in the Netherlands to 66.8% in Japan [49]. Recent studies worldwide have reported divergent prevalence of MRSA clones in different counties from the American continent [52], African counties [53], Middle East [54], Asia [55], and Europe [56]. This geographic variability of MRSA clones (e.g., community-associated vs. hospital-associated), may impact protein expression profiles and, ultimately, biomarker detection results.

### 4.1. Identification of PSM-Mec and Delta Toxin

The PSM-mec signal appeared consistently at 2414 ± 2 Da across studies [24,25,26,27,28]. This biomarker can only predict the presence of *mecA* gene in MRSA strains containing SCCmec type II, III, and VIII [24,25,26,27,28]. Moreover, it is not restricted to methicillin resistance in *S. aureus* but can also represent resistance in *S. epidermidis* [24,25,26,27,28,57,58]. Indeed PSM-mec had a specificity of 100% for methicillin resistance, but with low sensitivity and variable reproducibility, making this biomarker unreliable for detecting MRSA [24,25,26,27,28]. Josten et al. (2014) reported a sensitivity of 94.7%, which is higher compared to other studies [26]. Their results should be interpreted considering that they excluded *agr*-negative MRSA isolates with detectable PSM-mec, and only *agr*-positive PSM-mec isolates were counted for the assessment of the sensitivity [26]. The low sensitivity of PSM-mec can be explained by the variability in the expression of the *psm-mec* gene, occasionally generating small amounts of the peptide and by other peaks with greater intensity in the range of 3000–10,000 Da, mashing the biomarker [27,57,58]. In order to further increase the sensitivity of PSM-mec, some studies suggest optimizing detection threshold [24,25,26,27,28].

Detection of PSM-mec can only be achieved through intact cell protocols, preferably with ICM or O-PEM with formic acid [18,24,26,28,47]. Since this peptide is most likely washed away in the supernatant during the alcoholic solution in I-PEM protocol, its detection is rendered unfeasible [18,24,26,28,47].

PSM-mec expression depends on the functionality of the *agr* gene, a regulator gene [24,26,27,57,58]. The *agr* gene functionality can be assessed by the expression of delta toxin, detected in most isolates at 3006 ± 2 Da [24,26,27]. In MRSA, when PSM-mec signal is present, the delta toxin must also be present [24,26]. Josten et al. (2014), stated that delta toxin is also detectable at 3037 Da in CC1 strains [26]. This is due to a peak-shift from an allelic variant of delta toxin, which has a substitution of a glycine at position 10 by a serine, generating this *m*/*z* [59]. This biomarker can be detected by any sample protocol, but preferably with ICM and O-PEM [24,26]. The delta toxin has better sensibility than PSM-mec, presenting in some cases the highest peak intensity of the mass spectra, particularly in isolates with SCCmec II and III [24,26,27]. However, its presence is non-MRSA-specific, since it can be present in any *Staphylococcus* spp. *agr* positive; therefore, it does not by it itself indicate methicillin resistance [24,26,27].

In summary, these biomarkers have some diagnostic value but require support from additional biomarkers to improve the robustness of MRSA detection.

### 4.2. Additional Biomarkers and the Contribution of AI Tools

Edwards-Jones et al. (2000) and Du et al. (2002) arise as pioneer studies in the discrimination of MRSA and MSSA isolates by MALDI-TOF MS, as they identified further biomarkers besides PSM-mec and delta toxin [29,30]. However, differences in matrix preparation and the use of more basic data analysis limit comparability to more recent studies. Thus, greater emphasis should be placed on the biomarkers identified using the CHCA matrix solution and the current data analysis tools, in order to maintain the consistency of the results [9,60]. Especially considering that CHCA currently is the conventional matrix solution [9,60]. Additionally, Du et al. (2002), states that combination of an “algorithm method” with MALDI-TOF MS could be a promising strategy for bacterial identification [29].

Subsequent studies employed AI models to process divergent patterns through comparative mathematical and computational analysis of the PMF generated by MRSA and MSSA isolates. In general, AI models initially intervene in the pre-treatment of mass spectra by correcting the baseline, normalizing and smoothing the data [31,34]. Specifically for biomarker identification, AI models demonstrate the probability and diversity of mass spectral profiles, reducing the variability of complex datasets [21,22,31,32,33,34]. Additionally, according to the intrinsic criteria of each AI model, the biomarkers are categorized into MRSA and MSSA biomarkers, labeling the resistant and sensitive isolates [21,22,31,32,33,34]. For example, using the Principal Component Analysis, biomarkers can be grouped into features according to resistant and sensitive isolates in two- or three-dimensional graphical representations, enabling dimensional relationships to be established between samples. These models allowed selection of specific biomarkers related to the isolates’ resistance and sensitivity to methicillin, due to differences in biomarker intensity or biomarkers that did not overlap in PMF’s of MRSA and MSSA isolates.

Kim et al. (2019) stands out for employing twelve specific biomarkers for the discrimination of methicillin resistance, including biomarkers for SCCmec II, III, and IV, and MSSA, selected through a decision tree model which provided an identification of 87.6% of the isolates [31]. The selection of these biomarkers was carefully based on their high positive and negative predictive values, sensitivity and specificity [31]. In addition, there were avoided close *m*/*z* values, preventing misclassification of the biomarkers [31]. This study also considered the peak-shifts in the most suitable biomarkers, associated with specific *S. aureus* clones [31]. Also, this study did not disregard PSM-mec, since this biomarker still represents MRSA containing SCCmec type II, III, and VIII [24,25,26,27,28,57,58]. Additionally, biomarkers for SCCmec IV were included to address the rise in infections with community-associated MRSA, which have distinct phenotypic characteristics from healthcare-associated MRSA isolates [31,61,62].

Most of the remaining studies focused on differential expression or presence/absence of specific biomarkers. However, since some biomarkers may display variable or close peak intensities in MRSA and MSSA isolates, the lack of defined threshold values for peak intensity complicates classification. In Sogawa et al. (2017), the biomarker at 1935.9 Da showed average intensities of 880.8 and 662.2 for MRSA and MSSA, respectively [32]. Similarly, in Wang et al. (2013), the two biomarkers identified, at 3784 and 5700 Da may be present in MRSA and MSSA isolates, but only MRSA had higher peak intensity [33]. Lastly, in Abalkhail et al. (2022), Elbehiry et al. (2023) and Yu et al. (2022) presented biomarkers that were either present or missing in MRSA and MSSA isolates [21,22,34]. This highlights the need to standardize cut-off values for biomarkers, to ensure correct discrimination of methicillin resistance. Returning to Kim et al. (2019), the selected biomarkers still needed the definition of cut-off values [31]. Nonetheless, the presence of the twelve biomarkers lessens the impact of doubtful identifications, achieving higher robustness [31].

In Sogawa et al. (2017), it was selected a highly specific biomarker, identified as a penicillin-binding protein 2a (PBP2a) fragment [32]. As a remark, the *mecA* gene is not solely responsible for methicillin resistance in MRSA and intact PBP2a cannot be detected by this method, only its fragments are detected [4,23]. The *mecC* gene is a homolog of *mecA* gene prevalent in Europe that confers methicillin resistance, producing a protein similar to PBP2a that shows similar characteristics [4]. Most of the selected biomarkers in these studies did not correspond to an expressed protein. Whether any of the given biomarkers has some association with fragments of PBP2a expressed by *mecA* and *mecC* gene or any given methicillin resistance-related protein is not disclosed.

In many of these studies, a particular test sample and/or reference strains were selected in order to validate the designated biomarkers by a given AI model. The overall success rate for the differentiation of MRSA and MSSA was reported to be greater than 90% (Table 4) [21,22,29,30,31,32,33,34]. However, due to the lack of prevalence data, the accuracy presented is only an estimated value.

In these studies, investigators assembled large samples which generated a self-built database, besides the commercial database supplied by the equipment system. This can be a beneficial practice since it further increases the sample reference for discrimination [9,14,23]. Nonetheless, proper measures must be met in order to validate the database using a test sample. [9,23]. The test sample must be distinct from the sample assembled for biomarker selection and represent geographically diverse isolates to ensure generalizability [9,23]. Otherwise, this may lead to reduced precision despite the achievement in high sensitivity [30]. Using reference strains also contributes to accuracy since clinical isolates are affected by factors that may change phenotypic characteristics, unlike reference strains that are relatively phenotypically more stable and well known [9,23].

### 4.3. Alternative Protocols for MRSA Detection

Two studies introduced a novel protocol for direct MRSA detection from low-inoculum clinical samples and an optimized matrix solution.

Fan et al. (2024) proposed a method for detecting MRSA directly from whole blood and cerebrospinal fluid samples within 1.5 h by carrying out a pre-concentration and separation protocol using ampicillin-conjugated magnetic particles (AMP-MB) with subsequent resistance and sensitivity biomarkers discrimination by MALDI-TOF MS [35].

Since detecting microorganisms directly from biological samples requires centrifugation to decrease the interference of the sample matrix, the magnetic separation protocol reduced the influence of the biological matrix and maintained bacterial integrity [13,15,35]. Investigators stated that the capture efficiency of AMP-MB with MRSA was over 80% and the lower threshold of detection of MRSA in both samples was determined as 5 × 10^6^ CFU/mL [35]. This was fairly good, taking into account that other studies report that the sensitivity required for MALDI-TOF MS detection is approximately 10^4^ to 10^6^ CFU/mL [13,15]. However, ampicillin is a broad-spectrum antimicrobial [63]. Whereas this method was tested by co-inoculation of biological samples and phosphate-buffered saline solution with *Streptococcus pneumoniae* and *Klebsiella pneumoniae* isolates, stating that the detection was guaranteed as long as the proportion of MRSA was greater than 50% [35]. Nonetheless, this imposes a limitation in polymicrobial infections.

Additionally, Gao et al. (2022) used an esterified CHCA matrix, which was stated to have a higher performance in the extraction of hydrophobic peptides compared to the conventional matrix solution containing only CHCA [36]. This study demonstrated that the use of this matrix enabled the detection of discriminating biomarkers of MRSA and MSSA, which had not been detected using the CHCA matrix [36]. However, biomarkers obtained through esterified CHCA cannot be directly compared with the remaining biomarkers selected in the other studies using conventional CHCA. Nonetheless, according to Calderaro and Chezzi (2024), the introduction of a more efficient matrix solution for extracting hydrophobic proteins (i.e., lipoproteins) could enhance microbiological identification at the strain/serotype level [9]. It could also significantly advance the application of MALDI-TOF MS in detecting generalized resistance and enabling direct identification from biological samples [9].

This systematic review was conducted in accordance with PRISMA 2020 guidelines and registered in the PROSPERO database. However, several limitations should be acknowledged. Only 15 studies met the inclusion criteria, representing a limited dataset that may weaken the statistical power and generalizability of the findings. There was also limited geographic diversity among the selected studies, since the majority originated in Asian countries, which may affect the applicability of the results to global contexts. A notable source of heterogeneity was the variability in pre-analytical conditions—such as sample preparation, bacterial growth media, and processing protocols—as well as differences in analytical methodologies used to identify biomarkers, which could impact the reproducibility and comparability of the results. Even though we presented the results of the performance tests of the identified biomarkers, due to the number of articles collected, no further analysis was carried out. We chose not to do so due to the possibility of obtaining a low-robustness meta-analysis. Additionally, some studies lacked clear reporting on the use of gold-standard methods for methicillin resistance detection or the application of statistical analysis, introducing further risk of bias. Lastly, most of the assessed studies did not establish suitable cut-off values for biomarkers with variable expression, limiting their diagnostic reliability.

## 5. Conclusions

MALDI-TOF MS technology is highly effective for identification of microbiological isolates and demonstrates promising potential in distinguish between methicillin-resistant and methicillin-sensitive *S. aureus* isolates. However, biomarkers such as PSM-mec and delta toxin are not sufficiently discriminating for the standalone detection of MRSA by MALDI-TOF MS. The integration of additional biomarkers, particularly when coupled with AI models, substantially increases the laboratorial performance of the identification and discrimination of methicillin resistance in *S. aureus*. In addition, the development of protocols capable of detecting MRSA from samples with reduced inoculum, along with the optimization of the matrix, may contribute to improving the detection of MRSA and other pathogens as well.

Nevertheless, further studies should be performed not only to establish clear, clinically applicable cut-off values for biomarker intensity but also to report statistically significant differences in order to perform construction of databases suitable to wider geographic locations, which are critical for ensuring global applicability and diagnostics precision. These studies must be supported by robust sensitivity and specificity data obtained from validation cohorts, which is crucial for translating these methods from basic research to routine clinical application technologies.

## Figures and Tables

**Table 1:** Inclusion and exclusion criteria for paper screening.

Inclusion Criteria	Exclusion Criteria
Articles published in English language.	Articles published in other languages.
Articles published until 27 July 2024. Free full-text access.	Abstract of congress, reports, reviews/state-of-the-art articles.
Articles reporting findings from any country.	Studies involving veterinary infections.
Original scientific articles on the topic:	Other infectious agents.
MRSA and MSSA human infections.	Identification of isolates by MALDI-TOF MS.
Identification of biomarkers of *S. aureus.*	

**Table 2:** Systematic review summary table of included studies.

Author	Sample	Study Location	Methodology			Results		
			Reference Method	MALDI-TOF Analyser	Sample Preparation	MRSA Biomarkers (Da)	MSSA Biomarkers (Da)	AI Models
Abalkhail & Elbehiry, 2022 [21]	22 MRSA and 26 MSSA	Saudi Arabia	*mecA* gene PCR	MALDI Biotyper (Bruker Daltonics)	I-PEM *^1^	5530, 6580, 6710, and 6820	2771, 2996, 3720, 4480, 4540, and 6310	PCA
Alksne et al., 2020 [24]	26 MRSA and 28 MSSA	Latvia	*mecA* gene PCR	Autoflex Speed MS (Bruker Daltonics)	I-PEM *^1^ O-PEM *^1^ ICM *^1^	ICM and O-PEM: PSM-mec (2414 ± 2) ICM, O-PEM, and I-PEM: delta toxin (3006 ± 2)	ND	NA
Du et al., 2002 [29]	35 MRSA and 41 MSSA	China	*mecA* gene PCR	linear MALDI-TOF MS (Micromass UK Ltd.)	O-PEM *^2^	Main peaks: 2413.01 and 2453.54	Main peaks: 2547.91, 2585.28, 2686.45, and 2723.17	NA
Edwards-Jones et al., 2000 [30]	7 MRSA and 7 MSSA	UK	PFGE and phage typing	Kompact MALDI 2 linear TOF MS (Kratos Analytical)	O-PEM *^2^	891, 1140, 1165, 1229, 2127, 2454, and 3045	2548 and 2647	NA
Elbehiry et al., 2023 [22]	197 MRSA and 129 MSSA	Saudi Arabia	Kirby–Bauer test	MALDI Biotyper (Bruker Daltonik)	I-PEM *^1^	3990, 4120, and 5850	Lack of resistance peaks	PCA
Fan et al., 2024 [35]	20 MRSA and 30 MSSA	China	Automated AST	VITEK MS (bioMérieux)	Amp-MB protocol O-PEM *^3,5^	4304.6889 (larger a.u. in MRSA) 3874.4304 and 6889	3041.2293 (larger a.u in MSSA)	NA
Gao et al., 2002 [36]	21 MRSA and 41 MSSA	China	*mecA* gene PCR and Kirby–Bauer test	Autoflex max TOF/TOF MS (Bruker Daltonics)	I-PEM *^4^	4821 and 9645	2306 and 2322 (larger a.u in MSSA)	PCA
Hu et al., 2019 [25]	241 MRSA and 106 MSSA	China	Kirby–Bauer test	MALDI-Biotyper (Bruker Daltonics)	O-PEM *^1^	PSM-mec (2413 ± 2)	ND	Clinpro Tools
Josten et al., 2014 [26]	356 *S. aureus* *^7^	Germany	*mecA* gene PCR and Kirby–Bauer test	MALDI-TOF MS Biflex II (Bruker Daltonic)	O-PEM *^1^	PSM-mec (CC5): 2415 ± 4 delta toxin: 3007 (most CC) and 3037 (CC1)	ND	NA
				VITEK MS (bioMérieux)	ICM *^5^			
Kim et al., 2019 [31]	320 *S. aureus* (database) 181 *S. aureus* (test sample)	Korea	*mecA* gene PCR	Microflex LT MALDI-TOF MS (Bruker Daltonics)	O-PEM *^1^	SCCmec IV: 5541 (+) and 5053 (−) PSM-mec (SCCmec III specific): 2410 and 4607 At least one: 1975, 2410, 3890, 4607, and 6594	2194, 2339, and 2631	BioNumerics (decision tree model)
Paskova et al., 2020 [27]	35 MRSA	Multicentred	ND	microFlex MS (Bruker Daltonics)	O-PEM *^1^	PSM-mec (2413 ± 3.00) and delta toxin (3006 ± 3.00)	ND	NA
Rhoads et al., 2016 [28]	137 MRSA and 146 MSSA 12 MRSA USA 100-USA1200	USA	*mecA* gene PCR	VITEK MS (bioMérieux) Bruker MicroFlex (Bruker Daltonics): USA isolates	ICM *^5^	PSM-mec (2415 ± 2.00)	ND	NA
Sogawa et al., 2017 [32]	50 MRSA and 50 MSSA (algorithm) 34 MRSA and 31 MSSA (test sample)	Japan	*mecA* gene PCR	Autoflex II TOF (Bruker Daltonics)	O-PEM *^1^	1888.1 (430.3 a.u.), 1935.9 (880.8 a.u.), 2867.9 (1490.9 a.u.), 3044.2 (20,061.4 a.u.) *^6^, and 4641.3 (260.0 a.u.)	1935.9 (662.2 a.u.), and 2760.3 (1230.1 a.u.)	NL-SVM
Wang et al., 2013 [33]	48 MRSA and 52 MSSA	China	*mecA* gene PCR	MALDI-Microflex (Bruker Daltonics)	I-PEM *^1^	3784 and 5700 (larger a.u. in MRSA)	3784 and 5700 (smaller a.u. in MSSA)	Clinpro Tools (Genetic algorithm)
Yu et al., 2022 [34]	4309 MRSA and 3949 MSSA (algorithm) 12,101 MS (external validation)	Taiwan	Automated AST	MicroflexLT MALDI-TOF MS (Bruker Daltonics)	O-PEM *^1^	6593.2	6550.0	LightGBM

*^1^—CHCA matrix solution with 50% acetonitrile and 2.5% trifluoracetic acid; *^2^—CMBT matrix solution (acetonitrile/methanol/distilled water (1:1:1, *v*/*v*/*v*), with 0.1% formic acid and 0. 01 M ether 18-crown-6); *^3^—Extraction with formic acid and VITEK-MS-CHCA matrix (#411071): matrix solution of CHCA with 20–30% acetonitrile, 20–30% ethanol, and 3–5% trifluoracetic acid; *^4^—matrix solution of CHCA-C3 with acetonitrile dissolved in water distilled (*v*/*v* = 1/1) with 2% trifluoracetic; *^5^—VITEK-MS-CHCA (#411071): CHCA matrix solution with 20–30% acetonitrile, 20–30% ethanol, and 3–5% trifluoracetic acid, without formic acid; *^6^—*m*/*z* corresponding to a PBP2a fragment; ^7^—two isolates of ST239 from Australia and Portugal. Abbreviations: ND—not described; NA—not applied; CMBT—5-Chloro-2-mercaptobenzothiazole; CHCA—α-cyano-4-hydroxycinnamic acid; FA—formic acid; ICM—intact-cell method; O-PEM—on-target protein extraction method; I-PEM—in-tube protein extraction method; A. U.—average intensity; PCA—Principal Component Analysis; NL-SVM—Non-linear Support Vector Machine; LightGBM—Light Gradient Boosting Machine; MS-mass spectra.

**Table 3:** Performance test results for PSM-mec.

Performance Test	Alksne [24]	Hu [25]	Josten [26]	Rhoads [28]	Paskova [27]
Sensitivity (%)	61 *^1^	60.2	94.7 *^3^	37	50/90 *^4^
Specificity (%)	100 *^1^	100	100 *^3^	100	100
Reproducibility (%)	87 *^1^	―	―	―	33–100 *^5^
Repeatability (%)	―	1.7/18.4 *^2^	―	―	―

*^1^—with ICM; *^2^—intra-batch and inter-batch repeatability, respectively; *^3^—results with agr-positive MRSA isolates; *^4^—isolates with SCCmec II and III, respectively; *^5^—inter-laboratory reproducibility.

**Table 4:** Performance test results for additional biomarkers.

Performance Test	Abalkhail [21]	Du [29]	Edwards-Jones [30]	Elbehiry [22]	Fan [35]	Gao [36]	Kim [31]	Sogawa [32]	Wang [33]	Yu [34]
Accuracy (%)	100	90.79 *^1^	85.71 *^1^	97.87 *^1^	96.0 *^1^	96.8 *^1^	87.6	89.0–100	ND	―
AUC	―	―	―	―	―	―	―	―		0.78–0.91

Abbreviations: ND—Not disclosed; *^1^—calculated using MedCalc^©^ Software [41].

## Data Availability

No new data were created or analyzed in this study.

## Appendix A

### Appendix A.1

**Table A1:** Checklist for analytical cross-sectional studies [40].

No.	Question
1	Were the criteria for inclusion in the sample clearly defined?
2	Were the study subjects and the setting described in detail?
3	Was the exposure measured in a valid and reliable way?
4	Were objective, standard criteria used for measurement of the condition?
5	Were confounding factors identified?
6	Were strategies to deal with confounding factors stated?
7	Were the outcomes measured in a valid and reliable way?
8	Was appropriate statistical analysis used?

### Appendix A.2

**Table A2:** Joanna Briggs Institute’s critical appraisal tools results for the 15 studies [40].

Study	1.	2.	3.	4.	5.	6.	7.	8.
Abalkhail [21]	Y	Y	Y	Y	Y	NA	Y	Y
Alksne [24]	Y	Y	Y	Y	Y	NA	Y	Y
Du [29]	Y	Y	Y	Y	Y	NA	Y	Y
Edwards-Jones [30]	Y	Y	UC	Y	Y	NA	Y	Y
Elbehiry [22]	Y	Y	N	Y	Y	NA	Y	Y
Fan [35]	Y	Y	N	Y	Y	NA	Y	Y
Gao [36]	Y	Y	Y	Y	Y	NA	Y	Y
Hu [25]	Y	Y	N	Y	Y	NA	Y	Y
Josten [26]	Y	Y	Y	Y	Y	NA	Y	Y
Kim [31]	Y	Y	Y	Y	Y	NA	Y	Y
Paskova [27]	Y	Y	UC	Y	Y	NA	Y	Y
Rhoads [28]	Y	Y	Y	Y	Y	NA	Y	Y
Sogawa [32]	Y	Y	Y	Y	Y	NA	Y	Y
Wang [33]	Y	Y	Y	Y	Y	NA	Y	UC
Yu [34]	Y	Y	N	Y	Y	NA	Y	Y

Abbreviations: Y: Yes; N: No; UC: Unclear; NA: Not Applicable.
